# Report of Committee on Foreign Degrees

**Published:** 1898-11

**Authors:** 


### Editorial.

### REPORT OF COMMITTEE ON FOREIGN DEGREES.

An abstract of the report of this committee, presented to the National Association of Dental Faculties at Omaha, is given upon another page of this number, and a careful reading of it is urged upon all who feel an interest in the reputation of the dental profession in the United States. Its preparation does credit to the chairman, Dr. Barrett, for it is the clearest presentation ever given upon this important subject. The constant charges made abroad against the dental educational institutions of this country find here a complete, and, it is hoped, a final answer.

The report as given is described as an abstract. It is this only in the sense of having some personal matters cut out. In other respects it is as presented to the Association of Dental Faculties. It would have been more satisfactory to the writer to have given it in full, but, for reasons not necessary to enlarge upon, it was deemed better to omit, for the present, certain portions which require further legal proof.

It is undoubtedly true that the immoral side of life would never be presented if it were not preceded by an immoral demand; and this is axiomatic, whether confined to individuals, communities, or professions. The sale of fraudulent diplomas has been a source of continued trouble for many decades. The demand for these, as the author of the report truly states, has never come from this side of the water; in fact, a diploma of this character would be utterly valueless here, for, under existing State laws, it could never be admitted to registration, and, without this, practice under it would be an impossibility. The demand comes from various quarters of civilization, or these fraudulent manufactories could not exist. It has been evident to dental educators in this country that, for some unexplainable reason, there is a desire to possess one of these diplomas, for the stream of applications requesting a diploma *in absentia* has been constant for many years. In recognized dental colleges these have invariably gone into the waste-basket. To those familiar with the laws governing practice in Europe this repeated demand is inexplicable. The desire, however, exists, and hence the reason for the nefarious traffic described in the report.

It was supposed that when the authorities succeeded in imprisoning the notorius Buchanan, and death ended his career, that all further attempts to issue these diplomas would have ceased, but the constant and ever-increasing demand found new sources of supply.

It has been difficult for foreign practitioners to understand why this traffic should continue undisturbed. The writer of the report makes this sufficiently clear, but it is doubtful whether the explanation will satisfy these critics. The European standards of legal restraint are quite different from those in force here, but it is questionable whether, with similar conditions prevailing there, these men could be convicted under any of the governments of continental

Europe. It is one thing to be morally convinced that a man is guilty of a crime and quite another thing to be able to prove it, and the proof, as the report states, is not to be had in this country.

Whether this can be secured abroad is doubtful. These diplomas probably never come under the examination of the police. They serve a purpose for office examination, and give a certain character to the practitioner with a class who fail, through ignorance of the subject, to be able to distinguish between the false and the true, and thus the man acquires a private reputation of having an American diploma and an American training.

It is very doubtful whether this traffic in degrees can be stopped unless our *confrères* abroad can devise some means to secure legal evidence. That every effort will be made by the committee of the Dental Faculties having the matter in charge is assured from the well-known character of the chairman. He should receive the earnest co-operation of all professional associations, as well as individuals.

In order that the work may proceed without delay, money will be necessary. The Association of Dental Faculties is pledged to do its part financially; but it should not be left for this body to raise all the funds required, for the entire dental profession has an equal interest in the suppression of this blot upon it and upon our country. The writer would suggest that the committee ask for contributions from all local and State organizations. This will require time, but time is demanded to secure evidence necessary to carry these men into court.

The appointment of advisory committees abroad by the Dental Faculties is a move in the right direction. Complaints are constantly being made that men are received in the senior year by colleges in this country upon certificates of no value. This must be exceedingly rare now, with the more exact knowledge prevailing of continental methods. The appointment of these local committees will, however, obviate all the difficulties connected with foreign languages and foreign institutions, for no applicant for matriculation from abroad can be received unless his certificate has the endorsement of this advisory committee. Until these committees are appointed and get into good working order there will necessarily be some confusion, and perhaps some injustice to individuals, hence ample notification should be given for a full knowledge of this salutary measure both in this and foreign countries.

The efforts of the Association of Dental Faculties have been given from its first organization to the purification of professional teaching, and the labor of the present year has been simply a continuation of this good work. It can be depended upon to carry dental education to the extremest limit attainable, and to equally rid the country of all pretenders, whether they issue irregular diplomas or prey upon a too gullible public in other directions.

